# MicroRNA-mediated mechanotransduction and chondrocyte differentiation in mesenchymal stem cells

**DOI:** 10.1080/19768354.2026.2623320

**Published:** 2026-02-04

**Authors:** Taehwan Kim, Yangming Wang, Nayoung Suh

**Affiliations:** a Department of Medical Science, Soonchunhyang University, Asan-si, Republic of Korea; b Institute of Molecular Medicine, College of Future Technology, Peking University, Beijing, People’s Republic of China; c Beijing Advanced Center of RNA Biology (BEACON), Peking University, Beijing, People’s Republic of China; d Southwest United Graduate School, Kunming, People’s Republic of China; e Department of Pharmaceutical Engineering, Soonchunhyang University, Asan-si, Republic of Korea; f Institute for Molecular Metabolism Innovation, Soonchunhyang University, Asan-si, Republic of Korea

**Keywords:** Mesenchymal stem cells, mechanotransduction, mechanosensitive microRNAs, chondrogenic differentiation, cartilage degeneration

## Abstract

Mesenchymal stem cells (MSCs) integrate mechanical information from their microenvironment to regulate lineage commitment. Through integrin-based adhesion, cytoskeletal tension, and nuclear deformation, mechanical cues are transduced into intracellular signals via conserved pathways such as integrin–FAK/Src, RhoA–ROCK, and Hippo–YAP/TAZ. These pathways not only regulate chromatin accessibility and transcriptional output but also induce characteristic changes in mechanosensitive microRNAs (miRNAs). Mechanical loading alters miRNA expression programs that modulate focal adhesion assembly, Rho GTPase activity, and SMAD or Wnt signaling, thereby refining the SOX9-centered transcriptional networks that drive MSC chondrogenesis. Physiological mechanical stimuli including dynamic compression, fluid shear, and controlled tensile strain promote chondrogenic differentiation by lowering actomyosin tension, restricting YAP/TAZ nuclear localization, and enhancing SMAD–SOX9 cooperation. Conversely, pathological changes in the pericellular matrix, such as reduced stiffness and increased permeability, disrupt mechanical filtering, impair force transmission, and destabilize cytoskeletal organization. These mechanical defects shift chondrocytes toward high-tension, YAP-active states that suppress matrix gene expression and hinder maintenance of the chondrogenic phenotype. Simultaneously, dysregulation of mechanosensitive miRNAs weakens negative regulation of inflammatory and catabolic pathways, contributing to extracellular matrix degradation and progressive cartilage degeneration. Although numerous mechanosensitive miRNAs have been identified, their mechanistic roles and context-specific regulation remain incompletely defined. A deeper understanding of how miRNAs integrate diverse mechanical cues is essential to elucidate MSC fate transitions and the mechanobiology of cartilage repair. Advances in single-cell mechanobiology, mechanically tunable culture systems, and miRNA-targeted modulation may ultimately yield diagnostic indicators of mechanical imbalance and new therapeutic strategies for restoring cartilage homeostasis.

## Introduction

Mesenchymal stem cells (MSCs) are multipotent cells that contribute to the maintenance, repair, and regeneration of diverse tissues (Linero and Chaparro 2014; Pittenger et al. 1999). Their differentiation potential is shaped by a coordinated interplay between biochemical factors and the physical properties of their surrounding microenvironments (Kilian et al. 2010; McBeath et al. 2004; Pei et al. 2023). Among regulatory influences, mechanical stimuli have emerged as fundamental determinants of MSC behavior (Engler et al. 2006; Kilian et al. 2010; Yang et al. 2016). MSCs continuously sense the mechanical landscape of their niche via integrin–based adhesion complexes, cytoskeletal tension, and nuclear deformation (Buxboim et al. 2014; McBeath et al. 2004; Zhao et al. 2021). Any changes in these structural features activate conserved mechanotransduction pathways, including integrin–focal adhesion kinase (FAK)/Src, RhoA–ROCK, and Hippo–YAP/TAZ, which convert external forces into intracellular signals that regulate chromatin accessibility, transcriptional activity, and ultimately lineage commitment (Engler et al. 2006; Kilian et al. 2010; McBeath et al. 2004; Yang et al. 2016). Thus, MSC fate decisions are tightly coupled to the mechanical characteristics of the extracellular and pericellular environment (Table 1).

**Table 1.** Mechanotransduction-associated miRNAs regulating the integrin–FAK/Src, RhoA–ROCK, and Hippo–YAP/TAZ signaling axes in MSCs.

miRNAs	Targets	Signaling pathways	Function in MSC	Reference
miR-326-5p	ITGA5		Mechanotransduction regulator	Zhang et al. 2021
miR-138	FAK	Integrin–FAK/SRC	Anti-osteogenic regulator	Eskildsen et al. 2011
miR-146a-5p	TRAF6 IRAK1		Anti-senescent regulator	Xiao et al. 2021
miR-124	Rho A	Rho A–ROCK	Pro-neurogenic regulator	Wang et al. 2016
miR-339-5p	YAP1	Hippo–YAP/TAZ	Hippo regulator	Li et al. 2022
miR-376b-3p	YAP1		Anti-osteogenic regulator	Huang et al. 2020
miR-21-5p	YAP1		Anti-apoptotic regulator	Ji and Wang 2024
miR-1263	MOB1		Anti-apoptotic regulator	Yang et al. 2020

MicroRNAs (miRNAs) provide an additional regulatory layer (Chen et al. 2004; Yekta et al. 2004) that governs multiple aspects of MSC identity, including cytoskeletal organization, stress adaptation, and lineage progression. Increasing evidence implies that mechanical cues also modulate miRNA expression, defining a distinct class of mechanosensitive miRNAs (Kumar et al. 2014; Schmitz et al. 2019; Son et al. 2013). Through these actions, mechanosensitive miRNAs function not only as downstream effectors but also as modulators that fine-tune the fidelity and amplitude of mechanotransduction signaling. Changes in matrix stiffness, geometric confinement, cytoskeletal tension, and mechanical loading remodel miRNA profiles in ways that influence focal adhesion (FA) assembly, RhoA activity, nuclear mechanotransduction, and mechano-responsive transcriptional networks. Through these actions, mechanosensitive miRNAs function not merely as downstream effectors but as modulators that fine-tune the fidelity and amplitude of mechanotransduction signaling (Dole et al. 2021; Guan et al. 2011).

Chondrogenic differentiation provides a particularly compelling biological context in which mechanical and miRNA-dependent regulation converge. Cartilage formation and homeostasis rely on finely balanced mechanical forces that maintain low cytoskeletal tension, suppress YAP/TAZ nuclear activity, and promote SRY-box transcription factor 9 (SOX9)-driven transcriptional programs (Dupont et al. 2011; Hallstrom et al. 2023; Karystinou et al. 2015; Woods et al. 2005). Mechanical cues modulate the activation of the SMAD2/3, SMAD1/5/8, and Wnt pathways, collectively guiding chondrogenic identity acquisition and maintenance (Praxenthaler et al. 2018). Within this mechanobiological landscape, miRNAs (including those of the miR-29 family, miR-24, and others) modulate key transcription factors, extracellular matrix (ECM) regulators, and cytoskeletal components that play essential roles in cartilage matrix production. Thus, mechanosensitive miRNAs are critical integrators that couple biophysical information to lineage-specific genetic programs (Dole et al. 2021; Guan et al. 2011; Peng et al. 2022).

Despite progress in understanding each individual component, the field lacks an integrated framework that explains how mechanotransduction pathways and mechanosensitive miRNAs operate in parallel, intersect, and jointly shape MSC fate. The goal of this review is to synthesize current knowledge on MSC mechanobiology, mechanosensitive miRNA networks, and their combined roles in regulating chondrogenic differentiation. A further aim is to examine how these systems become disrupted in osteoarthritis (OA), in which changes in mechanical environments and post-transcriptional regulation contribute to the loss of cartilage homeostasis. By integrating these perspectives, this review outlines a conceptual foundation for understanding how mechanical and miRNA-mediated processes coordinate cartilage development and how their breakdown may lead to degeneration and disease.

## Mechanical remodeling and force-responsive behaviors in MSCs

MSCs experience diverse mechanical inputs within tissue environments, and these cues drive cellular remodeling that shapes their mechanical state and functional potential (Engler et al. 2006). Substrate stiffness is a major determinant of MSC morphology and cytoskeletal organization; cells on stiff matrices develop prominent actin stress fibers, reinforced FAs, and elevated intracellular tension, whereas soft substrates promote rounded, low-tension states with reduced actomyosin contractility (Pek et al. 2010). These cytoskeletal configurations influence nuclear shape and chromatin accessibility, establishing mechanical contexts that bias lineage outcomes.

Mechanical signals additionally remodel adhesion patterns and cortical actin networks. Increased rigidity or geometric constraint enhances integrin engagement and promotes the assembly of mature, force-bearing adhesion structures. Fluid shear stress generated by interstitial flow reorganizes actin at the cell cortex and modulates mechanosensitive ion channels, while cyclic compressive loading alters hydrostatic and osmotic pressure and can induce transient chromatin compaction (Kim et al. 2014; Zhao et al. 2015). Tensile strain applied at defined magnitudes aligns actin fibers, modulates nuclear deformation, and influences stretch-dependent changes in chromatin organization (Tajik et al. 2016).

Importantly, MSCs do not merely adapt transiently to mechanical inputs; they can acquire persistent mechanical memory. Exposure to stiff environments induces long-lasting changes in cytoskeletal tension and nuclear mechanics that persist even after transfer to softer substrates, stabilizing lineage biases and altering responses to subsequent stimuli (Killaars et al. 2019; Yang et al. 2014). This memory is reflected in altered transcriptional responsiveness and sustained mechanical phenotypes that shape MSC fate decisions over time.

Mechanical cues can also modulate miRNA expression in MSCs, linking structural remodeling to post-transcriptional gene regulation (Wang et al. 2011). For example, miR-100-5p and miR-143-3p increase on stiff matrices or high-tension 3D hydrogels in a RhoA-dependent manner and bias cells toward osteogenic programs (Frith et al. 2018; Wu et al. 2019). miR-145 responds to geometric elongation and is upregulated in MSCs subjected to anisotropic constraints (Yeh et al. 2019), while miR-21 expression is induced by mechanical priming on rigid matrices and remains elevated even after transfer to soft environments, functioning as a molecular effector of mechanical memory (Li et al. 2017a). These examples demonstrate that structural remodeling and mechanical state transitions are closely coupled to changes in miRNA expression, which help convert physical cues into longer-lasting phenotypic outcomes.

Together, these force-responsive behaviors illustrate the mechanical plasticity of MSCs and provide the cellular context within which molecular mechanotransduction pathways and miRNA-dependent regulatory mechanisms operate. This mechanobiological landscape forms the basis for pathway-specific regulation, which is explored in the following section.

## miRNA-mediated regulation of core mechanotransduction pathways

**Figure 1.**
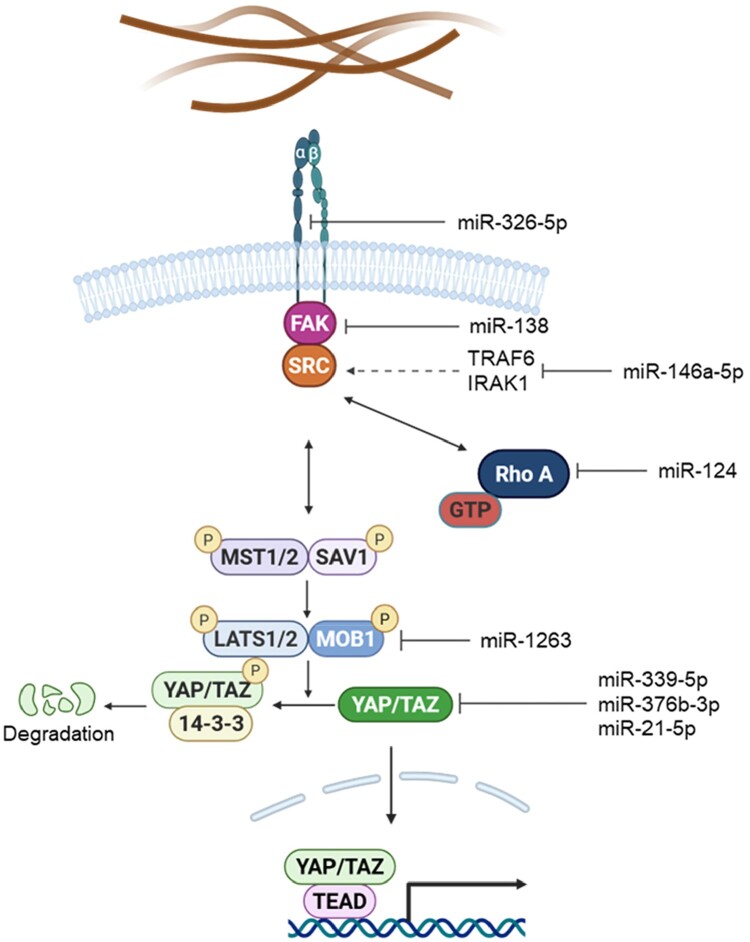
miRNA-mediated regulation of core mechanotransduction pathways in MSCs. Mechanical inputs transmitted through the extracellular matrix (ECM) are sensed by integrins, activating integrin–FAK/Src signaling and RhoA–ROCK–dependent cytoskeletal contractility. The resulting changes in actin tension and nuclear force transfer regulate the Hippo pathway, modulating YAP/TAZ phosphorylation, localization, and TEAD-dependent transcription. Mechanosensitive miRNAs modulate selected nodes within these pathways, such as focal adhesion (FA) components, RhoA activity, and YAP/TAZ stability, providing an additional post-transcriptional layer that fine-tunes mechanical signal output. Arrows indicate activation and blunt lines indicate inhibition. Created with BioRender.com.

Mechanical cues are decoded in MSCs through a limited set of conserved mechanotransduction pathways that regulate cytoskeletal architecture, intracellular tension, and mechanosensitive transcription (Salasznyk et al. 2007; Xu et al. 2012a). Among these, integrin–FAK/Src, RhoA–ROCK, and Hippo–YAP/TAZ signaling form three core axes that directly link ECM engagement and cytoskeletal state to changes in gene expression (Qiu et al. 2025; Salasznyk et al. 2007; Xu et al. 2012a). A growing body of evidence indicates that each of these pathways is subject to miRNA-dependent regulation at multiple nodes, providing a post-transcriptional layer that refines mechanotransductive output and influences MSC fate decisions (Hyvari et al. 2018; Wan et al. 2018) (Figure 1).

### Integrin–FAK/Src signaling

The integrin–FAK/Src pathway is the primary module through which MSCs translate ECM engagement into intracellular signaling (Sun et al. 2018; Zhou et al. 2021). Mechanical cues that increase integrin binding or clustering promote the formation of nascent FAs, where FAK undergoes autophosphorylation and recruits Src family kinases. The activated FAK–Src complex phosphorylates downstream adhesion components, facilitating FA maturation, actin polymerization, and the assembly of contractile actomyosin structures (Hyvari et al. 2018; Veevers-Lowe et al. 2011). These events strengthen force transmission to the cytoskeleton and nucleus, positioning the FAK/Src axis as a proximal regulator of intracellular tension and mechanosensitive transcription.

Targeted miRNA regulation provides an additional layer of control over this pathway. At the level of ECM engagement, miR-326-5p modulates cell–matrix interactions by repressing integrin α5, thereby limiting FA initiation (Zhang et al. 2021a). Downstream of integrin, miR-138 reduces FAK expression and phosphorylation, attenuating FA signaling and lowering cytoskeletal tension (Eskildsen et al. 2011), while miR-146a-5p attenuates Src activity by suppressing upstream regulatory kinases (Xiao et al. 2021). Through these coordinated interactions, miRNAs fine-tune the sensitivity and output of the integrin–FAK/Src module, enabling MSCs to adjust their mechanotransductive responses to changes in matrix stiffness or ligand density dynamically.

### RhoA–ROCK signaling

The RhoA–ROCK pathway serves as the central regulator of intracellular tension and force transmission within MSCs (Totsukawa et al. 2000). Mechanical cues that strengthen integrin engagement or restrict cell geometry promote the activation of RhoA, which transitions from its GDP-bound, inactive state to the active GTP-bound form. Activated RhoA stimulates Rho-associated kinase (ROCK), leading to phosphorylation of the myosin regulatory light chain and inhibition of myosin phosphatase (Grandy et al. 2022). These events reinforce actomyosin contractility, stabilize stress fibers, and enhance traction forces that are transmitted across the cytoskeleton to the nucleus. Through these effects, the RhoA–ROCK axis directly influences nuclear deformation, chromatin accessibility, and the activation of downstream mechanosensitive pathways (Kim et al. 2024).

miRNA-mediated regulation is a key mechanism modulating this tension-generating module. miR-124 directly targets RhoA, reducing RhoA–ROCK signaling and shifting MSCs toward low-tension mechanical states that support alternative lineage programs (Wang et al. 2016). By attenuating actomyosin contractility, miR-124 alters nuclear mechanics and downstream transcriptional responsiveness, illustrating how miRNAs fine-tune the mechanical set point of MSCs and influence their ability to interpret force-dependent cues (Li et al. 2016; Maharam et al. 2015).

### Hippo–YAP/TAZ signaling

The Hippo–YAP/TAZ pathway integrates signals from cytoskeletal tension, cell–matrix adhesion, and cell geometry to regulate transcriptional programs that control MSC proliferation and lineage specification (Bao et al. 2018). Low-tension states or restricted spreading activate the core Hippo kinases MST1/2 and LATS1/2, resulting in phosphorylation of YAP and TAZ, their binding to 14-3-3 proteins, cytoplasmic retention, and cytoplasmic degradation (Zhao et al. 2008). In contrast, elevated actomyosin contractility or culture on stiff substrates suppresses Hippo kinase activity, allowing unphosphorylated YAP and/or TAZ to accumulate in the nucleus, where they interact with TEAD transcription factors to promote mechanosensitive gene expression and influence MSC fate decisions (Miyoshi et al. 2023).

miRNAs also play a role. For example, miR-339-5p and miR-376b-3p directly target YAP1, reducing its expression and attenuating osteogenic differentiation in human bone marrow–derived MSCs (Huang et al. 2020; Li et al. 2022). Beyond intracellular regulation, MSC-derived exosomal miRNAs can modulate Hippo–YAP/TAZ signaling in recipient cells; for example, exosomal miR-27b-3p represses YAP1 in hepatic stellate cells, while miR-21-5p reduces YAP1 levels in cardiomyocytes (Cheng et al. 2023; Ji and Wang 2024). Upstream components of the Hippo cascade can also be miRNA-sensitive: miR-1263 targets MOB1, diminishing LATS activation and altering downstream YAP/TAZ activity (Chen et al. 2015a; Yang et al. 2020). Collectively, these interactions demonstrate how miRNAs fine-tune Hippo–YAP/TAZ signaling, shaping the transcriptional outcomes of mechanical cues in MSCs and related contexts.

## Mechanical regulation of chondrogenic differentiation and miRNA-dependent modulation

Mechanical stimuli have potent pro-chondrogenic effects on MSCs by reshaping cytoskeletal organization, modulating intracellular tension, and activating mechanoresponsive transcriptional programs (Ge et al. 2021; Mouw et al. 2007). Cyclic compressive loading, one of the most physiologically relevant forces in native cartilage, enhances the expression of SOX9, collagen type II alpha 1 chain (COL2A1), and aggrecan (ACAN) by promoting a rounded cell morphology, reducing actomyosin contractility, and stimulating signaling pathways that favor the production of cartilaginous matrix (Ge et al. 2021; Huang et al. 2005). The magnitude and frequency of compression are critical: moderate, intermittent loading generally elicits the most robust chondrogenic response, consistent with the dynamic mechanical environment of developing cartilage (Haugh et al. 2011; Li et al. 2010). Shear stress generated by perfusion or interstitial flow supports chondrogenesis by enhancing nutrient transport, promoting cytoskeletal alignment and reduced tension, and upregulating genes linked to matrix synthesis and chondroprogenitor identity (Kock et al. 2014; Schatti et al. 2011). Controlled tensile strain can further reinforce cartilage-like differentiation by suppressing hypertrophic transitions and maintaining SOX9 activity (Gong et al. 2025; Kanazawa et al. 2012).

These diverse mechanical inputs activate mechanotransduction mechanisms such as integrin–FAK/Src signaling, RhoA–ROCK modulation, nuclear deformation, and YAP/TAZ inactivation that collectively reduce cytoskeletal tension and establish a mechanical environment favorable for chondrogenic lineage commitment (Arnsdorf et al. 2009; Zhang et al. 2015). Across multiple experimental systems, the incorporation of mechanical stimulation accelerates matrix deposition, enhances the stability of the chondrocyte phenotype, and synergizes with biochemical cues such as TGF-β to drive robust chondrogenesis (Li et al. 2023; Xia et al. 2017).

**Figure 2.**
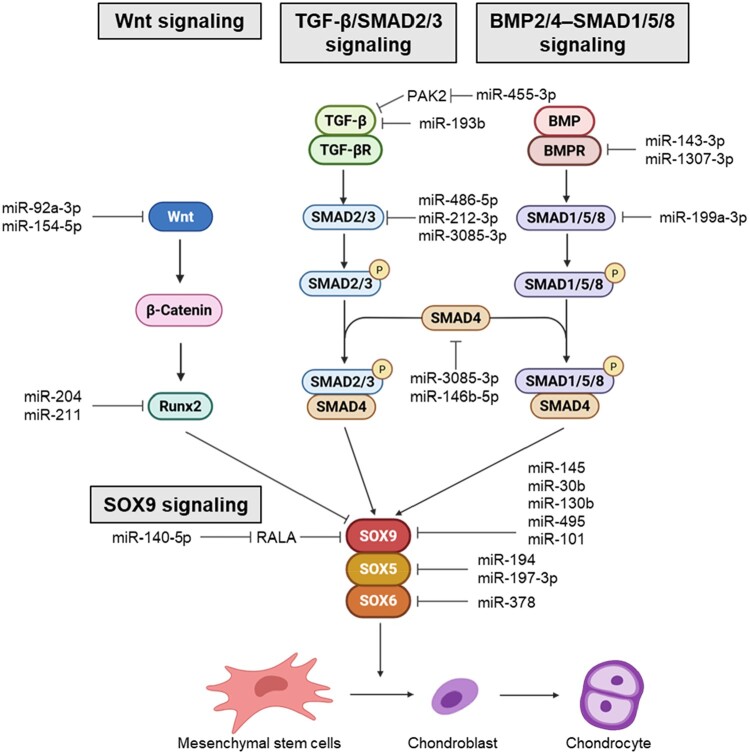
Signaling pathways and miRNA-mediated regulation of SOX9-driven chondrogenesis in MSCs. Schematic overview of the major pathways governing MSC chondrogenic differentiation. Wnt signaling pathways antagonize SOX9 through RUNX2, whereas the TGF-β/SMAD2/3 and BMP2/4-SMAD1/5/8 pathways activate SMAD complexes that promote SOX9, SOX5, and SOX6 transcription. Together, the SOX trio (SOX9, SOX5, SOX6) drives the expression of cartilage matrix genes such as COL2A1 and ACAN. Mechanosensitive and lineage-associated miRNAs target multiple nodes within these pathways, including Wnt components, SMADs, and the SOX trio, adding a post-transcriptional layer that modulates the balance between osteogenic and chondrogenic outcomes. Created with BioRender.com.

Within a mechanical environment conducive to differentiation, chondrogenic fate emerges through coordinated regulation across multiple signaling pathways rather than a single linear cascade. Mechanical cues bias the relative activities of Wnt, TGF–β–SMAD2/3, and BMP2/4–SMAD1/5/8 signaling toward SOX9–centered transcriptional programs, thereby shifting the balance from progenitor maintenance to chondrogenic commitment (Celik et al. 2019). Mechanosensitive miRNAs then fine-tune these pathways at multiple regulatory nodes – across receptors, intracellular mediators, and lineage transcription factors – thereby converting mechanical states into graded and temporally controlled signaling outputs that stabilize differentiation and limit inappropriate hypertrophic/osteogenic deviation (Figure 2, Table 2).

**Table 2.** miRNAs regulating Wnt, TGF-β/SMAD2/3, BMP2/4–SMAD1/5/8, and SOX9 signaling axes associated with chondrogenic fate determination in MSCs. Plus (+) and minus (−) symbols indicate promotive or inhibitory effects on chondrogenic differentiation, respectively. Upward (↑) and downward (↓) arrows denote upregulation or downregulation of the indicated miRNAs in osteoarthritis (OA), respectively.

miRNAs	Targets	Signaling pathways	Effect on chondrogenesis	Dysregulation in OA	Reference
miR-92a-3p	Wnt5a	Wnt	+	↓	Mao et al. 2018
miR-154-5p	Wnt11		+	↓	Li et al. 2015
miR-204	Runx2		+	↓	Huang et al. 2010
miR-211	Runx2		+	↓	Huang et al. 2019
miR-455-3p	PAK2	TGF-β/SMAD2/3	+	↓	Hu et al. 2019
miR-193b	TGF β		−	↑	Hou et al. 2015
miR-486-5p	SMAD2/3		−	↑	Liu et al. 2023
miR-212-3p	SMAD2/3		−	↑	Dai et al. 2024
miR-3085-3p	SMAD2/3		−	↑	Niu 2022
miR-3085-3p	SMAD4		−	↑	Niu 2022
miR-146b-5p	SMAD4		−	↑	Zhang et al. 2017
miR-3085-3p	SMAD4	BMP2/4–SMAD1/5/8	−	↑	Niu 2022
miR-146b-5p	SMAD4		−	↑	Zhang et al. 2017
miR-1307-3p	BMPR		−	↑	Yang et al. 2018
miR-143-3p	BMPR		−	↑	Yan et al. 2024
miR-199a-3p	SMAD1		−	↑	Lin et al. 2009
miR-140-5p	RALA	SOX9	+	↓	Karlsen et al. 2014
miR-145	SOX9		−	↑	Yang et al. 2011
miR-30b	SOX9		−	↑	Wa et al. 2017
miR-130b	SOX9		−	↑	Zhang et al. 2021
miR-495	SOX9		−	↑	Lee et al. 2014
miR-101	SOX9		−	↑	Dai et al. 2012
miR-194	SOX5		−	↑	Xu et al. 2012
miR-197-3p	SOX5		−	↑	Wang et al. 2023
miR-378	SOX6		−	↑	Feng et al. 2022

### SOX9 signaling pathway

The initiation and progression of MSC chondrogenesis are fundamentally driven by SOX9, a master transcription factor that orchestrates lineage commitment and cartilage-specific gene expression (Barter et al. 2017; Liao et al. 2014). SOX9 activates core ECM genes, including COL2A1, ACAN, and cartilage oligomeric matrix protein (COMP), and cooperates with SOX5 and SOX6 to establish a stable chondrogenic transcriptional program (Lefebvre et al. 1997; Liu and Lefebvre 2015). It also influences chromatin accessibility and suppresses hypertrophic transitions, thereby preserving early chondroprogenitor identity (Hata et al. 2013; Hattori et al. 2008). Sustained SOX9 activity is thus required both to initiate differentiation and support matrix deposition and maintenance of cartilage-like phenotypes throughout maturation (Bi et al. 2001; Haseeb et al. 2021).

Multiple miRNAs directly modulate this SOX9-centered network. miR-145 and miR-30b bind the SOX9 3′ UTR and repress its expression, attenuating TGF-β3–induced chondrogenic differentiation and reducing COL2A1, ACAN, COMP, and other matrix genes (Wa et al. 2017; Yang et al. 2011). Additional SOX9-targeting miRNAs, including miR-130b, miR-495, and miR-101, similarly inhibit chondrogenesis in MSC and BM-MSC models (Dai et al. 2012; Lee et al. 2014; Zhang et al. 2021b). In contrast, miR-140-5p indirectly reinforces SOX9 activity by targeting the small GTPase RAS-like proto-oncogene A (RALA), thereby enhancing SOX9 and ACAN translation and promoting matrix gene expression in human MSCs (Karlsen et al. 2014). Within the SOX trio, miR-194 directly targets SOX5, whereas miR-197-3p and miR-378 repress SOX5 and SOX6, respectively, in inflammatory chondrocyte or BMSC systems (Feng et al. 2022; Wang et al. 2023; Xu et al. 2012b). Collectively, these miRNAs control the initiation, amplitude, and stability of SOX9-centered transcriptional programs that are established by mechanical cues.

### Wnt signaling pathways

Wnt signaling has a dual, mechanically sensitive influence on MSC chondrogenesis (Diederichs et al. 2019; Du et al. 2016). Canonical Wnt/β-catenin signaling generally antagonizes early chondrogenic commitment by repressing SOX9 and promoting hypertrophic or osteogenic transcriptional programs (Akiyama et al. 2004; Hill et al. 2005). This inhibitory effect is amplified by high actomyosin tension or stiff matrices, which stabilize β–catenin and promote its nuclear accumulation (Samuel et al. 2011; Sen et al. 2022). In contrast, non-canonical Wnt pathways, including Wnt5a and Wnt11, help maintain a low-tension cytoskeletal state by regulating planar cell polarity, calcium signaling, and small GTPase activity, thereby indirectly limiting YAP/TAZ activity and supporting SOX9-driven transcription (Farrera-Hernandez et al. 2021; Gao et al. 2011).

Mechanically responsive Wnt pathways are further refined by miRNAs expressed in MSCs. miR-26b, which is downregulated in rats during MSC chondrogenesis, directly targets specific Wnt ligands and attenuates canonical β-catenin signaling; its loss enhances the expression of COL2A1 and ACAN (Huang et al. 2019b). miR–92a–3p, which is abundant in human MSC–derived exosomes, promotes cartilage formation by targeting WNT5A and biasing non-canonical Wnt signaling toward a pro-chondrogenic state (Mao et al. 2018). miR–140–5p, a hallmark miRNA of MSC chondrogenesis and a downstream effector of SOX9, modulates multiple components of the Wnt pathway, including the Frizzled receptor FZD6, thereby fine–tuning Wnt signaling responsiveness during the initiation of matrix production (Barter et al. 2015). In mechanically stretched adipose-derived MSCs, miR-154-5p directly suppresses WNT11, linking tensile forces to non-canonical Wnt/PCP regulation (Li et al. 2015). Beyond ligands and receptors, MSC-expressed miRNAs converge on RUNX2, a major β-catenin-responsive transcription factor that biases lineage toward hypertrophy or osteogenesis. miR-204 and miR-211 directly repress RUNX2, shifting the balance away from osteogenic outputs and reinforcing SOX9-centered chondrogenic identity (Huang et al. 2019a; Huang et al. 2010).

### TGF-β/SMAD2/3 signaling

The TGF-β/SMAD2/3 pathway is one of the most potent biochemical drivers of MSC chondrogenesis and is highly sensitive to the mechanical context (Allen et al. 2012; de Kroon et al. 2017). Soft matrices or reduced RhoA–ROCK tension enhance TGF-β receptor clustering and ligand responsiveness, resulting in robust phosphorylation of SMAD2/3 (Furumatsu et al. 2016; Melzer et al. 2024; Rys et al. 2015). Activated SMAD2/3 then translocate into the nucleus, where they cooperate with SOX9 as cartilage-specific enhancers to promote the transcription of COL2A1, ACAN, and other matrix genes while restraining hypertrophic or osteogenic programs (Chavez et al. 2017; Furumatsu et al. 2009; Furumatsu et al. 2005). High actomyosin tension, by contrast, can impair SMAD2/3 activation by changing receptor trafficking or nuclear mechanics, thereby weakening SOX9-mediated chondrogenesis (Chambers et al. 2018; Pfeifer et al. 2019).

miRNAs expressed in MSCs and chondrogenic progenitor models directly target multiple nodes in this cascade. miR-337-3p represses TGFBR2, diminishing upstream signaling strength, whereas miR-486-5p and miR-212-3p inhibit SMAD2 in BM-MSCs and related chondrocyte systems (Dai et al. 2024; Liu et al. 2023). miR-3085-3p suppresses SMAD3 and SMAD4, and miR-146b-5p targets SMAD4, collectively modulating the magnitude and duration of SMAD-dependent transcription (Niu 2022; Zhang et al. 2017). miR-199a-3p directly represses SMAD1, thereby influencing the balance between TGF-β/SMAD2/3 and BMP2/4–SMAD1/5/8 outputs during lineage specification (Lin et al. 2009). Beyond these direct interactions, several MSC–associated miRNAs fine–tune TGF–β/SMAD2/3 signaling through indirect mechanisms. miR–140–5p participates in a SMAD3–dependent regulatory loop during matrix production (Tardif et al. 2013), miR–21 modulates signals from TGF–β superfamily members such as growth differentiation factor 5 (GDF5) (Li et al. 2017b), and miR–455–3p together with miR–193b regulate upstream components including p21–activated kinase 2 (PAK2), TGF–β2, and TGFBR3 during MSC or chondroprogenitor differentiation (Hou et al. 2015; Hu et al. 2019).

### BMP2/4–SMAD1/5/8 signaling

The BMP2/4–SMAD1/5/8 axis is a complementary, mechanically sensitive pathway that can promote chondrogenesis in MSCs under low-tension conditions (Chen et al. 2015b). In rounded, low-tension cells, BMP receptor activation is more efficient, leading to strong SMAD1/5/8 phosphorylation; then, activated SMAD complexes translocate to the nucleus and enhance the gene expression of early cartilage markers, expanding the pool of SOX9-positive progenitors, increasing chromatin accessibility, and promoting transcription of cartilage-specific genes (Brauer et al. 2024; Chan et al. 2022). Under stiff or high-tension conditions, however, RhoA–ROCK activation is favored and BMP-driven chondrogenesis is reduced, with lineage trajectories diverted toward alternative outcomes (Gegg and Yang 2020; Selig et al. 2020).

Several miRNAs directly tune this BMP–SMAD module. In BM-MSCs, miR-143-3p and miR-1307-3p both target BMPR2; overexpression of either miRNA lowers BMPR2 levels, and in the case of miR-1307-3p reduces SMAD1/5/8 phosphorylation, proteoglycan deposition, and COL2A1 and ACAN expression during chondrogenic differentiation (Yan et al. 2024; Yang et al. 2018). Other miRNAs modulate BMP-driven outputs more indirectly. For example, miR-520d-5p promotes chondrogenesis by targeting HDAC1, thereby enhancing chromatin accessibility and increasing COL2A1 and ACAN expression (Lu et al. 2020; Tian et al. 2018). miR-199a-3p binds the SMAD1 3′ UTR in mesenchymal/chondroprogenitor models, antagonizing BMP2-induced SMAD1 activation and attenuating BMP-driven chondrogenesis even when ligand and receptors are present (Lin et al. 2009). Other miRNAs modulate BMP2/4–SMAD1/5/8 signaling outputs more indirectly. For example, miR-140-5p, which is strongly induced during chondrogenesis, targets regulatory factors including DNPEP and SMAD1 in chondrocytes and supports COL2A1 and ACAN induction in human MSC pellets (Miyaki et al. 2010).

Although TGF-β/SMAD2/3 and BMP2/4–SMAD1/5/8 activate distinct transcriptional programs, their effectiveness is fundamentally constrained by mechanical state (Aprile et al. 2022). Nuclear deformability, chromatin accessibility, and cytoskeletal tension determine how efficiently SMAD complexes engage cartilage enhancers and cooperate with the SOX trio (Furumatsu et al. 2005). Low-tension environments promote stable SMAD–SOX9 interactions and robust chondrogenesis, whereas high tension redirects SMAD outputs toward proliferative or hypertrophic programs (Dong and Jin 2025; Woods and Beier 2006). Mechanotransduction-associated miRNAs such as miR-140, miR-92a, and miR-455 provide feedback by tuning SMAD expression, receptor sensitivity, and matrix assembly (Chen et al. 2024; Hecht et al. 2019; Li et al. 2018; Zheng et al. 2024). Through these integrated mechanisms, SMAD complexes act as biochemical interpreters of mechanical information, ultimately converging on the SOX9 axis to secure a stable chondrogenic fate in MSCs.

## Mechanical deterioration and miRNA dysregulation in cartilage

The maintenance of cartilage homeostasis depends on a finely tuned mechanical microenvironment that preserves chondrocyte mechanosensing capacity and supports SOX9-centered transcriptional programs (Nishino et al. 2010; Sakai et al. 2009). When joints experience chronic overload, traumatic injury, or age-dependent matrix deterioration, this mechanical equilibrium becomes destabilized (Chery et al. 2020; Danalache et al. 2019; Felka et al. 2016). Structural degeneration of the pericellular matrix (PCM), which may include loss of ACAN, fragmentation of COL2A1, and disruption of proteoglycan networks, reduces PCM stiffness and alters force-distribution properties, leaving chondrocytes exposed to unbuffered mechanical stresses rather than the filtered, physiologic loading provided by intact cartilage (Alexopoulos et al. 2005a; Alexopoulos et al. 2005b; Lee et al. 2023). In parallel, increased PCM permeability amplifies fluid flow and osmotic fluctuations, disrupting the balance between hydrostatic pressure, cytoskeletal tension, and mechanosensitive ion-channel activity that chondrocytes rely on to interpret mechanical cues (Darling et al. 2010; Kroupa et al. 2023; Wilusz and Guilak 2014).

Together, reduced stiffness and elevated permeability compromise the mechanical filtering system that maintains chondrocyte stability and bias the cellular mechanical state toward one incompatible with sustained chondrogenic transcription. As a result, core mechanotransduction pathways including integrin–FAK/Src, RhoA–ROCK, and Hippo–YAP/TAZ become dysregulated (Whitney et al. 2012; Xu et al. 2016; Zhang et al. 2016b). These perturbations weaken cytoskeletal integrity, destabilize FAs, impair nuclear mechanosensing, and shift the intracellular tension landscape toward a high-ROCK, YAP-active state that antagonizes SOX9. In this context, YAP/TAZ no longer remains appropriately inhibited and exhibits persistent or aberrant nuclear localization, reducing the transcription of COL2A1 and ACAN and undermining the matrix-producing identity of chondrocytes (Li et al. 2021 ). Concurrent activation of NF-κB and induction of catabolic enzymes such as MMP13 and ADAMTS5 further erode ECM integrity, creating a feedforward deterioration loop (Tetsunaga et al. 2011; Yang et al. 2023).

Disruption of mechanosensitive miRNA networks compounds these mechanical defects. miRNAs that ordinarily reinforce chondrogenic transcriptional stability, such as miR-140, miR-29, miR-24, and miR-146a, are consistently downregulated in osteoarthritic cartilage (Chaudhry et al. 2022; Guan et al. 2018; Karlsen et al. 2016; Le et al. 2016; Philipot et al. 2014). Loss of these miRNAs removes critical negative regulation of inflammatory cascades, derepresses matrix-degrading enzymes such as ADAMTS5, and weakens the post-transcriptional support required to maintain SOX9 activity. Age-related reductions in mechanosensitive miRNAs further diminish the ability of chondrocytes to adapt to fluctuating mechanical conditions and accelerate progression toward biomechanical and transcriptional instability (Si et al. 2017; Toury et al. 2022).

**Figure 3.**
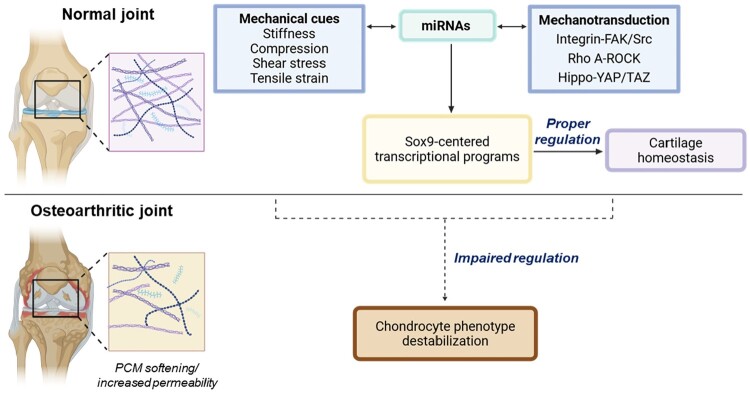
Integrated control of cartilage homeostasis by mechanical cues and mechanosensitive miRNAs. Upper panel, physiological mechanical cues, including matrix stiffness, compression, shear stress, and tensile strain, are sensed by chondrocytes and transduced through core mechanotransduction pathways such as integrin–FAK/Src, RhoA–ROCK, and Hippo–YAP/TAZ signaling. Mechanosensitive miRNAs act as bidirectional modulators between mechanical inputs and intracellular signaling networks, fine-tuning cytoskeletal tension and transcriptional outputs. These integrated signals converge on SOX9-centered transcriptional programs that maintain cartilage homeostasis. Lower panel, disruption of mechanical integrity and miRNA regulation destabilize SOX9 signaling, leading to impaired cartilage homeostasis and progression toward osteoarthritis (OA). Solid arrows indicate intact regulatory signaling, whereas dashed arrows denote impaired regulation under pathological conditions. Created with BioRender.com.

These findings collectively indicate that cartilage degeneration is not driven solely by biochemical or inflammatory insults but arises from a coordinated breakdown of the mechanical and post-transcriptional systems that maintain chondrocyte identity (Figure 3). Mechanical cues fail to propagate correctly due to PCM deterioration; mechanotransduction pathways shift toward a tension-dominant, YAP-active state; and miRNAs that normally stabilize the chondrogenic program become dysregulated. The convergence of these defects collapses the integrative mechanotransductive–transcriptional framework that supports cartilage homeostasis (Agarwal et al. 2021; Bian et al. 2012; Zhang et al. 2016a).

This mechanobiological perspective implies new therapeutic opportunities, such as restoring physiological mechanical environments, correcting aberrant mechanotransductive signaling, and re–establishing mechanosensitive miRNA networks that reinforce SOX9 activity and matrix production (Hattori et al. 2021; Iseki et al. 2019). Approaches that simultaneously target these interconnected axes may be particularly effective in re-stabilizing the chondrocyte phenotype and halting cartilage degeneration.

## Conclusions

Mechanical cues, mechanotransduction pathways, and miRNA-mediated regulatory networks operate as an integrated system that governs MSC behavior, chondrogenic lineage specification, and long-term cartilage homeostasis. Across a range of experimental and physiological contexts, MSCs interpret changes in matrix stiffness, compression, shear stress, and intracellular tension through conserved signaling axes including integrin–FAK/Src, RhoA–ROCK, and Hippo–YAP/TAZ, and translate these mechanical inputs into SOX9-centered transcriptional programs that drive chondrogenesis. Mechanosensitive miRNAs add an essential post-transcriptional dimension to this framework by tuning adhesion dynamics, cytoskeletal organization, SMAD and Wnt signaling, and the stability of chondrogenic gene regulatory networks. Together, these multilayered mechanisms define how mechanical information is encoded and stabilized into cell fate decisions.

Conceptually, MSC chondrogenesis is best understood as a tightly regulated balance between competing signaling axes. Wnt signaling activity often counteracts SOX9–driven differentiation, whereas TGF–β/SMAD2/3 and BMP2/4–SMAD1/5/8 pathways promote chondrogenic commitment and matrix production. The mechanical state biases the relative dominance of these pathways. Mechanosensitive miRNAs reinforce this balance by acting across multiple layers that include receptors, SMAD/Wnt intermediates, and lineage transcription factors, thereby buffering pathway cross-talk and stabilizing chondrogenic identity under fluctuating biophysical cues.

Although this framework highlights critical regulatory axes, important mechanisms remain unresolved. The causal links between specific mechanical stimuli, miRNA regulation, and downstream lineage outcomes are still incompletely resolved, in part because most mechanosensitive miRNAs have been identified in reductionist *in vitro* systems that do not mimic the complex biophysical environments of developing or degenerating cartilage. The impact of PCM mechanics, especially softening and altered force transmission during early degeneration, on miRNA expression and mechanotransductive fidelity remains poorly understood. In addition, few studies have systematically compared how different mechanical modalities, such as stiffness, dynamic compression, shear flow, or tensile strain, uniquely reconfigure miRNA networks and mechanotransductive signaling. At the systems level, an integrated ‘mechanotransduction–miRNA–lineage’ framework that connects mechanical sensing with transcriptional stabilization has yet to be fully mapped.

Future research should focus on identifying mechanosensitive miRNAs across well-defined mechanical landscapes, incorporating single-cell and spatial transcriptomic profiling under controlled loading, and resolving the temporal dynamics by which miRNAs coordinate early mechanosensing events with later differentiation programs. Integration of biophysical measurements of ECM and PCM mechanics with miRNA-based regulatory analyses are essential to understand how mechanical deterioration contributes to cartilage pathology. Such efforts may ultimately reveal miRNAs that serve as sensitive indicators of mechanical imbalance or as therapeutic entry points for restoring chondrogenic signaling in degenerative joint disease.

Recognizing the interplay between these miRNA categories will provide an increasingly refined framework for interpreting how mechanical environments shape both early mechanotransductive responses and later differentiation-associated stabilization. By unifying mechanical cues, signaling pathways, and post-transcriptional regulation into a cohesive model, this review underscores the importance of mechanosensitive miRNAs as central mediators that couple mechanical environments to chondrocyte identity. Continued exploration of these interactions holds considerable promise for advancing our understanding of cartilage biology and for developing mechanistically informed strategies to diagnose, prevent, and treat musculoskeletal disorders.
